# Supplementary material for: Puzzles in modern biology. IV. Neurodegeneration, localized origin and widespread decay
Source: F1000Res. 2016 Oct 19;5:2537. [Version 1] doi: 10.12688/f1000research.9790.1 (PMC5288670; doi:10.12688/f1000research.9790.1)
Supplement: Peer review history

# Peer review: Referee response for version 1

**DOI:** 10.5256/f1000research.10555.r17108

**Published:** 2016-11-22

**Authors:** Anya Plutynski

**Affiliations:** 1 Department of Philosophy, Washington University St. Louis, St. Louis, MO, USA

I had only few minor comments and a suggested reference:  

- First, there was some redundancy in the paper: local v. spatially distributed origins as possibilities was mentioned at least twice.
- Second, I really liked the following point, and wondered if this might be developed further, “mechanistic studies often do not explicitly frame analysis of cause in terms of the variety of potential mechanisms for local triggers and spread versus the variety of potential mechanisms for dispersed origin. My only purpose here is to clarify the relation between different mechanisms and the broader framework in which we must understand the puzzles of disease onset and progression.” The idea here seems to be that mechanistic approaches must always be supplemented or contextualized in specific ways, if our aim is to differentiate between alternative hypotheses about origins of disease? Perhaps a firmer and more general statement to this effect might be worth making. As a general point, this is worth emphasizing!
- Last: are local v. spatially distributed initiations of the disease mutually exclusive options for a given disease, or could one be in play in some cases, and another be in play in other cases? To explain, perhaps in some cases a local lesion may advance so quickly that it clearly is a single origin story. In other cases (perhaps for other cancer types or subtypes), typically, many independent populations of porto-cancer cells may be arising simultaneously. For cancers of epithelial origin, the latter seems likely; Martincorena *et al.,* (2015) provide independent evidence in favor of the notion that mutations are always accumulating in healthy tissue, most epithelial cells slough off and do not become cancers, but some acquire sufficient mutations to advance to disease.

I have read this submission. I believe that I have an appropriate level of expertise to confirm that it is of an acceptable scientific standard.

## References

[1] MartincorenaIRoshanAGerstungMEllisPVan LooPMcLarenSWedgeDCFullamAAlexandrovLBTubioJMStebbingsLMenziesAWidaaSStrattonMRJonesPHCampbellPJ: Tumor evolution. High burden and pervasive positive selection of somatic mutations in normal human skin. *Science*.2015;348(6237) : 10.1126/science.aaa6806 880-6 10.1126/science.aaa6806 25999502PMC4471149

# Peer review: Referee response for version 1

**DOI:** 10.5256/f1000research.10555.r17107

**Published:** 2016-11-08

**Authors:** Christos Proukakis

**Affiliations:** 1 Department of Clinical Neuroscience, Institute of Neurology, University College London, London, UK

In this topical and well argued paper, Steve Frank proposes the framework within which one should consider causes of disparate disease processes as either primarily local, or primarily generalized. The focus is mainly on neurodegeneration, and specifically ALS. This condition does appear to start in one location, at least clinically, and spread in an apparently contiguous manner in most cases. A helpful contrast is drawn between the possibilities of a local event which may include a somatic mutation (on which he has eloquently written in the past) [1], and spread, which is an idea that has certainly spread over recent years. Importantly, the agents of spread may not be proteins in all cases, and this is stated here, as RNA and others are also mentioned.

 One point that deserves some discussion is the assertion that somatic mutations would generally be limited to causing local onset. This is true if one accepts that a single cell, or handful of neighbouring cells, could be the trigger. A more widespread onset, but still due to somatic mutations, could be caused by widespread dispersion of early somatic mutations. There is now clear evidence of extensive mixing of cells in early development, with work particularly from the Chris Walsh lab showing how a somatic mutation in brain could also be present throughout a broad region, and perhaps even further afield at a lower level.[2] Apparently synchronous or near-synchronous onset in disparate locations could be due to such a phenomenon, with multifocal onset reported in Parkinson’s disease.[3] This would lead to spatially separated positions progressing independently as stated, but the origin could still be a somatic mutation, which happened early enough in development to allow its progeny to be spatially separated.

 It is implicit that neurodegenerative diseases affect specific brain cell types, which differ in each one, with selective vulnerability being a key determinant of pathology, along with initiation / spread process. Most readers will be aware of this, but this could be explicitly clarified e.g. to page 2 “the broad neural landscape”.

 “Trigger versus spread”. I note the suggestion that an inherited mutation, present therefore in all cells, may lead to earlier onset by allowing spread to start earlier. This is in line with a staging pathology scheme proposed in a genetic subset of ALS, which claims to describe the sequence of spread.[4] I do not conceptually understand why one has to invoke spread in situations where every cell carries a mutation which can act in a direct or indirect pathogenic way locally. If the mutation has a pathogenic effect, then surely no spread is required when it is present in all cells? I accept that this does not exclude spread, which could still underlie a temporal sequence of events, but differential vulnerability could also determine the sequence of events, particularly if it is stereotyped.

 The author finally proceeds to compare this potential dichotomy with cancer and cardiovascular diseases, as situations where, after local initiation, pathology can spread through relevant factors. While it may seem obvious, it should be stated that in the case of cancer the agent of spread is the cell, which clearly is not the case in neurodegenerative disorders. Intriguingly perhaps one could actually invoke the cell as the agent of spread of somatic mutations through migration at the early neurodevelopmental stage, resulting in the spatially disparate somatic mutation situation I outlined above. Furthermore, the medium through which distant spread occurs in these disease categories, circulating blood, does not appear relevant to neurodegeneration, which (if spread is involved) would have to spread through physically connected neurons. Whether this occurs through trans-synaptic spread, secretion (which may or may not involve exosomes), or tunneling nanotubes, is an ongoing debate, albeit beyond the scope of this valuable opinion piece.

I have read this submission. I believe that I have an appropriate level of expertise to confirm that it is of an acceptable scientific standard.

## References

[1] FrankSA: Evolution in health and medicine Sackler colloquium: Somatic evolutionary genomics: mutations during development cause highly variable genetic mosaicism with risk of cancer and neurodegeneration. *Proc Natl Acad Sci U S A*.2010;107 Suppl 1: 10.1073/pnas.0909343106 1725-30 10.1073/pnas.0909343106 19805033PMC2868288

[2] LodatoMAWoodworthMBLeeSEvronyGDMehtaBKKargerALeeSChittendenTWD'GamaAMCaiXLuquetteLJLeeEParkPJWalshCA: Somatic mutation in single human neurons tracks developmental and transcriptional history. *Science*.2015;350(6256) : 10.1126/science.aab1785 94-8 10.1126/science.aab1785 26430121PMC4664477

[3] DicksonDWFujishiroHDelleDonneAMenkeJAhmedZKlosKJJosephsKAFrigerioRBurnettMParisiJEAhlskogJE: Evidence that incidental Lewy body disease is pre-symptomatic Parkinson's disease. *Acta Neuropathol*.2008;115(4) : 10.1007/s00401-008-0345-7 437-44 10.1007/s00401-008-0345-7 18264713

[4] BrettschneiderJDel TrediciKToledoJBRobinsonJLIrwinDJGrossmanMSuhEVan DeerlinVMWoodEMBaekYKwongLLeeEBElmanLMcCluskeyLFangLFeldengutSLudolphACLeeVMBraakHTrojanowskiJQ: Stages of pTDP-43 pathology in amyotrophic lateral sclerosis. *Ann Neurol*.2013;74(1) : 10.1002/ana.23937 20-38 10.1002/ana.23937 23686809PMC3785076
